# Changes in Circulating Procalcitonin Versus C-Reactive Protein in Predicting Evolution of Infectious Disease in Febrile, Critically Ill Patients

**DOI:** 10.1371/journal.pone.0065564

**Published:** 2013-06-06

**Authors:** Sandra H. Hoeboer, A. B. Johan Groeneveld

**Affiliations:** 1 Department of Intensive Care, VU University Medical Center, Amsterdam, The Netherlands; 2 Department of Intensive Care Medicine, Erasmus Medical Center, Rotterdam, The Netherlands; University of Leicester, United Kingdom

## Abstract

**Objective:**

Although absolute values for C-reactive protein (CRP) and procalcitonin (PCT) are well known to predict sepsis in the critically ill, it remains unclear how changes in CRP and PCT compare in predicting evolution of: infectious disease, invasiveness and severity (e.g. development of septic shock, organ failure and non-survival) in response to treatment. The current study attempts to clarify these aspects.

**Methods:**

In 72 critically ill patients with new onset fever, CRP and PCT were measured on Day 0, 1, 2 and 7 after inclusion, and clinical courses were documented over a week with follow up to Day 28. Infection was microbiologically defined, while septic shock was defined as infection plus shock. The sequential organ failure assessment (SOFA) score was assessed.

**Results:**

From peak at Day 0–2 to Day 7, CRP decreased when (bloodstream) infection and septic shock (Day 0–2) resolved and increased when complications such as a new (bloodstream) infection or septic shock (Day 3–7) supervened. PCT decreased when septic shock resolved and increased when a new bloodstream infection or septic shock supervened. Increased or unchanged SOFA scores were best predicted by PCT increases and Day 7 PCT, in turn, was predictive for 28-day outcome.

**Conclusion:**

The data, obtained during ICU-acquired fever and infections, suggest that CRP may be favoured over PCT courses in judging response to antibiotic treatment. PCT, however, may better indicate the risk of complications, such as bloodstream infection, septic shock, organ failure and mortality, and therefore might help deciding on safe discontinuation of antibiotics. The analysis may thus help interpreting current literature and design future studies on guiding antibiotic therapy in the ICU.

## Introduction

In critically ill patients, new onset fever often prompts clinicians to search for nosocomial microbial infection and to start empiric antibiotics in attempts to diminish morbidity and mortality [1]–[3]. Plasma levels of C-reactive protein (CRP) or procalcitonin (PCT) are often used to increase the a priori probability of microbial infection and sepsis in the intensive care unit (ICU) [2]–[12]. In a previous study on absolute levels within the first days after new fever onset in critically ill patients, we found that CRP may particularly help in predicting local microbial infection and PCT in predicting bloodstream infection (BSI) and a downhill clinical course [3]. The clinical relevance of changes in the markers is less clear, however. Changes in CRP and PCT over 2–7 days have been described in non-critically ill patient populations [13]–[20], in relatively small studies, about 50 patients or less [4], [9], [13], [15], [16], [19], [21]–[25], in specific conditions [2], [8], [10], [13], [15]–[17], [19], [20], [22], [24], [26]–[29] or in heterogeneous conditions in the ICU [4]–[7], [9], [11], [21], [23], [30]–[32], to judge the course of infection and its sequelae. CRP decreases of more than about 25% per day within the first week of treatment of (bloodstream) infections or sepsis have been suggested to help predict a beneficial response and disease course, while slower decreases or increases have been associated with persistent infection, organ failure or mortality, also in the ICU [7,8,11,17–19,21-,23,26–29,32]. A relatively rapid fall of PCT may be associated with a beneficial outcome of pneumonia, meningitis, burn-associated or other infections, whereas a rise may be associated with organ failure and mortality and thus might have predictive value (5,6,8–10,15,16,20,24,26,27,30,31]. A fall in PCT below 0.5 ng/mL, a threshold suggested in studies on non-critically ill patients, has been used to safely and effectively shorten the duration of administration of antibiotics for infections in the ICU [33]. CRP and PCT changes have been compared in their relative ability to detect the evolution of infection or sepsis, within [4]–[6], [10], [24]–[27], [30], [31] or outside the ICU [8], [14], [15], [18], [19]. CRP may display slower kinetics than PCT and in some, but not all studies, decreases or increases of the latter may better predict a beneficial or downhill course with resolving or aggravating organ failure, respectively [2], [4], [6], [8], [10], [14]–[16], [19], [24]–[27], [30], [31], [34]. Nevertheless, the relative value of marker changes in predicting response to antibiotic treatment of ICU-acquired infections is only rarely addressed [10], [15], [24], [26], [27], [31]. Hence, general conclusions on the relative superiority of the markers for specific endpoints in the critically ill are hard to draw from the literature and the controversy is ongoing.

In order to further help clinical decision making on the basis of changes in CRP and PCT in the ICU, we hypothesized for the current study that, in general critically ill patients with new onset fever, the 1-week course of CRP and PCT levels can be used to distinguish resolving microbial infection with a beneficial outcome from non-resolving or developing infection with a detrimental outcome associated with BSI, septic shock, organ failure and death. We also hypothesized that CRP and PCT differ in this respect, so that CRP primarily predicts the course of local infection and PCT that of systemic infection and its adverse sequelae. This would also help to define values at which antibiotic treatment can be decided as appropriate or to allow safe discontinuation in the ICU.

## Materials and Methods

This is a prospective observational study on causes and consequences of ICU-acquired fever, approved by the medical ethical committee of the VU University Medical Center, Amsterdam, conducted between 2003 and 2007. All patients or closest relatives gave their written informed consent, leading to inclusion of 101 consecutive patients with new onset fever, admitted to a mixed medical/surgical ICU, and the complete protocol has been described elsewhere [3]. The current analysis is on the 72 patients having completed a follow up of at least 7 days in the ICU (Consort diagram Figure S1). To briefly reiterate, a body temperature >38.3°C measured rectally was the main inclusion criterion, while admitted to the ICU for at least 24 hours without fever (body temperature <37.5°C). Exclusion criteria were: age under 18 years, pregnancy and life expectancy of <24 hours. Enrolment had to be completed within 12 hours of meeting inclusion criteria and was marked Day 0 (D0). Demographic data were collected. Disease severity was expressed through the simplified acute physiology scores (SAPS) II and monitored using sequential organ failures assessment (SOFA)-scores. On Days (D) 0, 1, 2 and 7 clinical data were recorded and blood was drawn for determination of routine parameters and markers. Antibiotic treatment and changes within the study period were recorded.

Routine chest- and sinus-radiographs were taken on D0 and 7, all other diagnostic imaging were ordered by treating physicians, blinded to results, as considered necessary. Blood samples for microbiological culture were taken from indwelling arterial catheters using delayed vial entry bottles for aerobic and anaerobic cultures and processed according to protocol. Depending on suspicion of local infection, specimens for microbial culture were collected. Investigation of fungal, viral or atypical microorganisms was left at the treating physician’s discretion. All culture and staining results from specimens drawn between D0–7 were evaluated. Positive cultures considered to represent colonization were not considered to represent infection and microorganisms are grouped according to genus. The presence of infection was determined by researchers (SHH and ABJG) blinded to study results and classified by likelihood into possible, probable or proven infection, according to criteria defined at the International Sepsis Forum Consensus Conference [35]. Infections are only considered when probable or proven [3]. Sepsis was defined as infection in the presence systemic inflammatory response syndrome (SIRS) criteria, according to ACCP/SCCM (American Society of Chest Physicians/Society of Critical Care Medicine) [36]. Criteria of shock criteria were a systolic arterial pressure <90 mmHg or a mean arterial pressure (MAP) <70 mmHg for at least one hour, despite fluid resuscitation, or need of vasopressor treatment. Shock in the presence of sepsis was marked septic shock. Organ failure was assessed by the sequential organ failure assessment (SOFA) score on Day 0, 1, 2 and 7.

Patients were divided into four categories of infectious course: Group 1 with infection presenting at D0–2 and without evidence of infection at D3–7, thus having a response to treatment and resolving infection, Group 2 with infection presenting D0–2 and persisting positive cultures from the same infection site and/or persisting positive cultures with the same microorganism at D3–7, thus having treatment failure, Group 3 without infection D0–2 and with infection D3–7, thus having a new infection, and Group 4 without infection D0–2 and D3–7. Complications of infection were considered BSI, septic shock and outcome. Therefore a similar division was done for BSI, irrespective of local cultures. Group 1a with BSI presenting D0–2 and without BSI D3–7, thus resolving BSI in response to treatment. Group 2a with BSI presenting D0–2 and with persisting BSI D3–7, i.e. treatment failure, Group 3a without BSI D0–2 and with infection D3–7, having a new BSI and Group 4a without BSI D0–2 and D3–7. Septic shock was defined by the presence of shock within 12 hours prior or 12 hours after the determination of infection in either one of the respective study intervals. Group 1b represents presence of septic shock in D0–2 but without septic shock in D3–7, Group 2b presents septic shock both in D0–2 and D3–7, Group 3b absence of septic shock in D0–2 but presence of septic shock D3–7, and Group 4b absence of septic shock in both study intervals. Analogously, we divided patients with decreasing SOFA scores (peak D0–2 to 7; Group 1c), unchanged SOFA scores (Group 2c) and increasing SOFA scores (Group 3c). Outcome is 28-day survival or all-cause mortality.

Routine parameters measured were white blood cell count (WBC) (Sysmex SE-9000 analyzer, Toa Medical Instruments, Kobe, Japan, normal values 4.5–10 x10^9^/L), lactate (Enzymatic method, Modular analytics <P> Roche diagnostics, Mannheim, Germany, normal values <1.8 mmol/L) and C-reactive protein (CRP, Immunoturbidimetric assay, Modular analytics <P> Roche diagnostics, Mannheim, Germany, normal values <5 mg/L). Procalcitonin was measured through the Kryptor compact system (Brahms Diagnostica, Henningsdorf, Germany, normal values <0.08 ng/L) using time resolved amplified cryptate emission (TRACE) technology.

### Statistical Analysis

**Figure 1:**
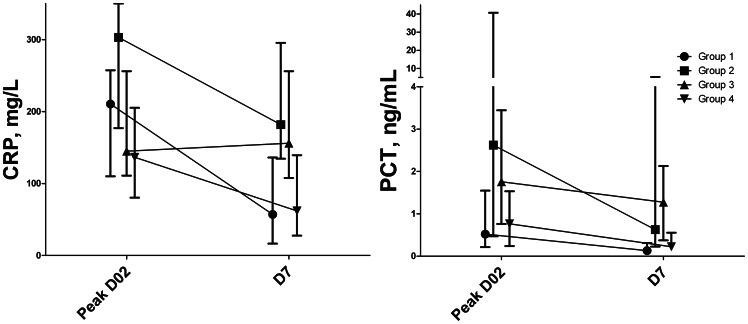
Evolution of C-reactive protein and procalcitonin according to evolution of infection (I) in febrile critically ill patients. CRP and PCT levels presented as median (interquartile range). Group 1=I Day (D)0-2 no I D3-7; Group 2=I D0-2 and I D3-7; Group 3=no I D0-2 but I D3-7; Group 4=no I D0-2 nor D3-7. For CRP D0-2 P=0.009, for CRP D7 P=0.002, for change P=0.004; for PCT D0-2 P=0.054, PCT D7 P<0.001, for change P=0.23, among groups.

CRP and PCT courses were expressed as fractional changes at D7 vs. peak values at D0–2. To further separate differences in absolute levels and changes, we used the Kruskal-Wallis test to evaluate group differences in the respective values. Area under the receiver operating characteristic curves (AUROC) were used to evaluate predictive values, such as sensitivity and specificity of optimal cut off values, defined at highest combined sensitivity and specificity, and their statistical significance. Exact P values are given unless <0.001, and values <0.05 were considered statistically significant. Data are expressed as number (percentage) or median (range) in tables and median, interquartile range (IQR) in Figure 1.

## Results

Table 1 represents patient characteristics and Table S1 infection characteristics, grouped according to course of infection. Fifty-two patients with new onset fever had resolving (Group 1) or no (Group 4) infection and only twenty had non-resolving (Group 2) or new (Group 3) infection, but (change of) treatment was mostly successful and ultimate mortality did not differ among groups. Most patients with infections had SIRS and therefore sepsis. The number of days from ICU admission until study inclusion was lowest in Group 2 and 3 and the need for vasopressor and renal replacement therapy was highest in Group 3. Infections with yeast were more persistent than infections with other microorganisms. 28-Day mortality in BSI was for Group 1a-4a 13, 100, 40 and 16%, respectively (P = 0.02), and in septic shock for Group 1b–4b 19, 40, 20 and 17%, respectively (P = 0.68).

**Table 1:** Patient characteristics.

		Group 1	Group 2	Group 3	Group 4	P
		n = 30	n = 9	n = 11	n = 22	
**General**						
Age (year)		62 (19–79)	63 (36–71)	68 (22–76)	66 (26–77)	0.91
Gender (male)		22 (73)	6 (68)	8 (73)	16 (73)	0.98
SAPS II on admission		48 (21–72)	49 (29–78)	50 (24–84)	45 (27–85)	0.99
Peak SOFA at D0–2		8 (3–13)	8 (3–13)	10 (4–14)	8 (3–16)	0.31
SOFA at D7		5 (0–15)	5 (4–14)	7 (5–13)	5 (2–21)	0.28
Temp °C	D0–2	39.2 (38.5–40.8)	39.3 (38.6–40.0)	39.0 (38.7–40.0)	39,0 (35,8–39,8)	0.80
	D7	37.6 (36.5–39.0)	39,2 (36,7–40,0)	38,1 (37,3–39,7)	37,8 (36,5–40,0)	0.03
Change		0,96 (0,89–1,00)	1,00 (0,93–1,02)	0,98 (0,94–1,03)	0,96 (0,93–1,00)	0.05
SIRS,	D0–2	30 (100)	9 (100)	11 (100)	22 (100)	1.0
	D7	23 (85)	9 (100)	9 (82)	17 (90)	0.65
Sepsis,	D0–2	30 (100)	9 (100)	0 (0)	0 (0)	1.00
	D7	0 (0)	9 (100)	9 (82)	0 (0)	0.19
Septic shock,	D0–2	15 (50)	6 (67)	0 (0)	0 (0)	0.39
	D7	0 (0)	7 (78)	8 (73)	0 (0)	0.80
Days from admission to D0		7 (1–77)	4 (1–12)	5 (1–16)	12 (2–78)	0.01
Duration mechanical ventilation, days						
		22 (5–82)	26 (4–42)	16 (11–82)	35 (10–123)	0.15
ICU length of stay, days		28 (10–95)	26 (12–44)	26 (12–85)	37 (11–126)	0.41
28-day mortality		5 (17)	2 (22)	2 (18)	5 (23)	0.95
ICU mortality		7 (23)	1 (11)	2 (18)	5 (23)	0.87
**Admission category**						
Trauma		4 (13)	1 (11)	2 (18)	4 (18)	0.94
Surgery						
general		17 (57)	6 (67)	9 (82)	13 (59)	0.50
cardiac		1 (3)	1 (11)	1 (9)	3 (14)	0.60
vascular		2 (7)	1 (11)	4 (36)	9 (2)	0.08
Neurologic		5 (17)	2 (22)	1 (9)	3 (14)	0.86
Respiratory insufficiency		17 (57)	2 (22)	4 (36)	7 (32)	0.16
post CPR		3 (10)	0 (0)	2 (18)	0 (0)	0.18
Sepsis		12 (40)	3 (33)	1 (9)	7 (32)	0.32
Shock		9 (30)	1 (11)	0 (0)	7 (32)	0.13
**Treatment up to 7 days prior to inclusion**						
Corticosteroids		14 (47)	3 (33)	4 (36)	12 (55)	0.65
SDD		8 (27)	2 (22)	4 (36)	12 (55)	0.65
Surgery		4 (13)	2 (22)	1 (9)	2 (9)	0.77
**Treatment during study D0–7**						
Antibiotics		30 (100)	9 (100)	11 (100)	19 (86)	0.07
Change in antibiotics		20 (67)	8 (89)	7 (64)	13 (59)	0.46
Corticosteroids		19 (63)	4 (44)	4 (36)	12 (55)	0.65
SDD		8 (27)	2 (22)	4 (36)	12 (55)	0.65
Mechanical ventilation		29 (97)	8 (89)	11 (100)	22 (100)	0.35
Inotropic/vasopressors		16 (57)	8 (89)	11 (100)	13 (59)	0.03
Renal replacement therapy		1 (3)	0 (0)	4 (36)	2 (9)	0.01
Surgery		3 (10)	1 (11)	2 (18)	3 (14)	0.91

Median (range), or number (percentage); SAPS = simplified acute physiology score; SOFA = sequential organ failure assessment score; ICU = intensive care unit; CPR = cardiopulmonary resuscitation; SDD = selective decontamination of the digestive tract. Group 1 =  infection (I) Day (D)0–2 not D3–7; Group 2 =  I D02 and I D3–7; Group 3 = no I D0–2 but D3–7; Group 4 = no I D0–2 nor D3–7.

### Course of Infectious Disease, CRP and PCT

Figure 1 shows the differences in marker levels in time for the infection groups. Table 2 shows that, among changes, those in CRP, rather than PCT, differed between groups according to infectious status, with a large decrease in Group 1 and persistently high values in Group 2 and 3, whereas absolute values of both CRP and PCT differed among groups. Table S2 shows that, among changes, those in PCT best discriminated between BSI groups, whereas absolute values of WBC, PCT and lactate at D7 differed among groups. Table S3 displays course in time for septic shock showing that, among changes, those in PCT better discriminated between groups than changes in CRP, whereas absolute values of WBC (D7), CRP, PCT also differed among groups. Table S4 shows that changes in SOFA score were particularly associated with changes in PCT.

**Table 2:** Evolution of infection.

	Group 1	Group 2	Group 3	Group 4	p
	n = 30	n = 9	n = 11	n = 22	
WBC D0–2, x10^9^/L	13.9 (2.5–24.4)	12.8 (7.9–81.7)	16.2 (9.0–24.8)	11.6 (7.8–23.9)	0.22
WBC D7, x10^9^/L	11.5 (4.9–23.2)	16.9 (8.3–30.2)	16.2(6.4–33.0)	11.9 (5.3–29.2)	0.06
WBC change	0.80 (0.47–3.20)	0.75 (0.37–1.41)	1.16 (0.40–2.95)	0.94 (0.50–1.65)	0.55
CRP D0–2, mg/L	210 (5–397)	303 (102–421)	145 (38–440)	137 (27–248)	0.009
CRP D7, mg/L	57 (2–267)	182 (22–416)	156 (49–304)	62 (6–265)	0.002
CRP change	0.40 (0.02–1.15)	0.68 (0.07–1.82)	0.93 (0.44–6.97)	0.58 (0.11–2.58)	0.004
PCT D0–2, ng/mL	0.5 (0.08–45.1)	2.6 (0.08–75.3)	1.7(0.3–6.3)	0.8 (0.1–2.8)	0.054
PCT D7, ng/mL	0.1 (0.06–38.5)	0.6 (0.1–24.3)	1.3 (0.3–20.8)	0.2 (0.08–4.3)	<0.001
PCT change	0.30 (0.05–1.57)	0.42 (0.04–2.97)	0.52 (0.08–68.3)	0.44 (0.11–5.88)	0.23
Lactate D0–2, mmol/L	1.6 (0.5–3.5)	1.5 (0.9–3.5)	1.3 (1.0–2.3)	1.4 (0.5–2.0)	0.60
Lactate D7 mmol/L	1.1 (0–4.3)	1.1 (0.9–3.1)	1.1 (0.7–1.8)	1.0 (0.5–2.2)	0.67
Lactate change	0.77 (0–2.08)	0.95 (0.48–1.72)	0.85 (0.43–1.60)	1.06 (0.42–1.50)	0.32

Median (range) for WBC = white blood cell count; CRP = C-reactive protein; PCT = procalcitonin; Group 1 = infection.

(I) Day (D)0–2 not D3–7; Group 2 = I D0–2 and I D3–7; Group 3 = no I D0–2 but D3–7; Group 4 = no I D0–2 nor D3–7.

### Predictive Values for Evolving Infectious Disease

Table 3 summarizes the predictive values of changes in markers for resolving (Group 1, a, b or c) or new (Group 3, a, b, or c) infection, BSI, septic shock or organ failure vs. other groups. Most AUROC’s were above 0.70, with high specificities of optimal cut off values. The table shows that CRP changes particularly predict changes in the status of infections and their complications knowingly BSI and septic shock, whereas PCT changes primarily predict the latter complications and the course of organ failure. Optimal cut off values are shown with decreases in markers by 86% or more and increases by 23% or more, and higher sensitivities of PCT than of CRP.

**Table 3:** Predictive values for changes of markers.

Infection	Resolving (Group 1)			New (Group 3)	
	Cut off	AUROC	P	Sens	Spec	PPV	NPV	Cut off	AUROC	P	Sens	Spec	PPV	NPV
WBC change	–	–	–	–	–	–	–	–	–	–	–	–	–	–
CRP change	<0.14	0.72	<0.001	31	93	75	65	>2.57	0.76	<0.001	20	100	100	88
PCT change	–	–	–	–	–	–	–	–	–	–	–	–	–	–
Bloodstream infection														
	Resolving (Group 1a)							New (Group 3a)						
	Cut off	AUROC	P	Sens	Spec	PPV	NPV	Cut off	AUROC	P	Sens	Spec	PPV	NPV
WBC change	–	–	–	–	–	–	–	>2.57	0.87	<0.001	20	98	50	94
CRP change	<0.04	0.73	0.04	29	57	50	92	>2.95	0.84	<0.001	20	100	100	94
PCT change	–	–	–	–	–	–	–	>2.00	0.89	<0.001	60	97	60	97
Septic shock	Resolving (Group 1b)							New (Group 3b)						
	Cut off	AUROC	P	Sens	Spec	PPV	NPV	Cut off	AUROC	P	Sens	Spec	PPV	NPV
WBC change	–	–	–	–	–	–	–	–	–	–	–	–	–	–
CRP change	<0.06	0.70	0.01	31	98	83	83	>2.57	0.83	<0.001	20	100	100	81
PCT change	<0.13	0.72	0.007	31	93	56	83	>1.78	0.82	<0.001	50	97	71	92
SOFA scores	Not increasing (Group 1c+2c)							Not decreasing (Group 2c+3c)						
	Cut off	AUROC	P	Sens	Spec	PPV	NPV	Cut off	AUROC	P	Sens	Spec	PPV	NPV
WBC change	–	–	–	–	–	–	–	–	–	–	–	–	–	–
CRP change	–	–	–	–	–	–	–	>2.95	0.67	0.02	6	100	100	78
PCT change	–	–	–	–	–	–	–	>1.23	0.73	0.001	38	92	60	83

WBC = white blood cell count; CRP = C-reactive protein; PCT = procalcitonin; AUROC = area under the receiver operating characteristic curve; Sens = sensitivity; Spec = specificity at optimal cut off values. PPV = positive predictive value; NPV = negative predictive value. Statistically significant AUROC’s are given only. A value less than 1 denotes a fractional decrease from Day 0–2 to 7 and a value above 1 a fractional increase.

### D7 Values: Group Differences and Outcome Prediction

At D7, CRP had a cut off <20.9 mg/L predicting Group 1 (at AUROC 0.67, P = 0.01, sensitivity 31 and specificity 93%). For PCT, the cut off was <0.18 ng/mL (at AUROC 0.76, P<0.001, sensitivity 63 and specificity 93%). For a PCT cut off <0.5 ng/mL sensitivity was 87 and specificity 45%. PCT at D7 also predicted, at a cut off >7.8 ng/mL (AUROC 0.67, P = 0.03, sensitivity 13 and specificity 96%), no decrease in SOFA score (Group 3c+2c). On D7, only WBC and PCT were lower in 28-day survivors than in non-survivors (P = 0.003 and 0.02, respectively). The AUROC for non-survival for WBC at D7, at an optimal cut off >33.1 x10^9^/L, was 0.76 (P<0.001, with sensitivity of 0 and specificity of 100%) and for PCT at D7 0.70 (P = 0.005, with sensitivity 29 and specificity 95%), at an optimal cut off >2.6 ng/mL.

## Discussion

This prospective, medium-sized study shows that changes in CRP and PCT, rather than changes in WBC, in general critically ill patients with new onset fever predict infectious courses in response to treatment, but in a different manner. CRP changes appear most predictive for changes in infectious status and complications such as BSI and development of septic shock. Whereas PCT changes primarily appear predictive for infectious complications BSI, development of septic shock, associated organ failure and death.

Our study is the third one comparing CRP and PCT and the second one comparing their courses in the evolution of nosocomial infection in the general critically ill patient with new onset fever [9], [12]. Our data do not suggest different kinetics per se over a 5–7 day course, in contrast to the slower kinetics of CRP than of PCT in the critically ill suggested by others [4], [6], [8], [10], [12], [15], [27], [31], [34]. This study however, suggests that CRP changes are more associated with evolution of infection and PCT more with evolution of adverse infectious sequelae. Although the AUROC’s of changes in markers seem higher than those for absolute levels, our current findings on absolute levels are in line with those reported earlier [3], suggesting that during nosocomial fever in the critically ill CRP is more likely to contribute to infection diagnosis whereas PCT has better capability of predicting risks of infections. PCT has also been reported to be capable, more than CRP, of early discrimination between [severe] infection or sepsis on the one hand and non-infectious SIRS or uncomplicated infection on the other [2], [4]–[6], [8]–[10], [12]. PCT increases predicted bloodstream invasion, septic shock and organ failure and carried greater prognostic significance in the course of infectious disease than CRP [on D7] thus supporting that PCT is more useful in predicting infectious complications, also in the ICU, even when not predicting new BSI [2], [4]–[6], [8]–[10], [12], 14,20,24–27,30,31. In leptospirosis, PCT normalises upon treatment in 4 days and CRP within 7 days in non-severe cases and both return to normal in 7 days in severe infections [19], suggesting greater sensitivity of PCT than of CRP to infection severity, in line with our data. Conversely, we can assume in line with others [10], [15], [17], [21]–[23], [28], [29], [31], [32], that the decrease in CRP in Group 1 with resolving infection resulted from appropriate antibiotic treatment, so that we cannot exclude that the persistent infection in Group 2 with less decreases was caused by treatment failure or slow response, even though not associated with increased mortality. The threshold for CRP at D7 to decide on the resolution of infection only is about 21 mg/L. Our study does not agree with the relation between rate of decline in CRP up to D7 during treatment for infection in the ICU and survival [22], [24], [26]–[29], [31], since the change of CRP did not predict outcome. The difference with our study may relate to differences in inclusion criteria, among others. Our study suggesting greater value of decreases in CRP than of PCT in resolving nosocomial infection in the ICU, does also not agree with the reported superior value of PCT decreases in predicting response of infections to treatment [10], [15], [26], [31]. A decrease of PCT to 0.5–1.0 ng/mL or lower has otherwise been used for allegedly safe discontinuation of antibiotics in patients with presumed infection given antibiotics with high likelihood for survival in the ICU [8]–[10], [12], [16], [24], [26], [27], [31], [33]. However, our data are not in line with this threshold and suggest a lower value of about 0.2 ng/mL, after one week treatment, since the values associated with non-resolving infection, increasing SOFA and mortality are higher.

Some limitations of this study should be addressed. A fair number of patients received corticosteroids, including so called low dose steroids for treatment of relative adrenal insufficiency during sepsis, prior and during inclusion, but this may hardly affect marker levels as related to the course of infection as our data in line with those of others suggest [13], [17], [37]. We used selective decontamination of the digestive tract by non-absorbable antibiotics for infection prevention in many of our patients but the use apparently did not confound the value of CRP and PCT [changes]. We cannot exclude that Group 4 patients without infections had benefited from this type of infection prevention and had received overtreatment by empiric antibiotics, in the absence of demonstrable microbial infection. We chose to classify all groups similar to our primary outcome variable: course of infectious disease, thus always entailing four groups per outcome measure, except for SOFA score. This resulted in uneven numbers per patient group, and the small number of patients in group 2a is a consequence of this uniform categorisation. However, small groups may not invalidate statistical significance. The study carries the advantage over many others [2], [4]–[7], [17], [30]–[32] of documentation of microbial infection and definitions of infectious complications [rather than stages of ‘sepsis’] in the critically ill. Finally, our study lacks daily measurements, as other have done in spite of increasing costs [2], [5]–[11], [14], [15], [18], [20], [21]–[23], [25], [31], [32], so that we cannot conclude on rapid time courses of the infection markers. However, our data suggest clinical usefulness of the sampling regimen followed. We did not evaluate biomarker changes over shorter periods since the kinetics of resolving and developing infections may differ according to infectious focus and causative microorganism, among others.

In conclusion, our study on ICU-acquired fever and infections suggests that CRP may be favoured over PCT courses over 5–7 days in judging response to antibiotic treatment, whereas the latter may better indicate the risk of complications, such as bloodstream infection, septic shock, organ failure and mortality, which may help deciding on safe discontinuation of antibiotics. The analysis may thus help interpreting current literature and design future studies on guiding antibiotic therapy in the ICU.

## Supporting Information

Figure S1 Consort diagram.
(PDF)

Table S1 Infection characteristics.
(DOCX)

Table S2 Evolution of bloodstream infection.
(DOCX)

Table S3 Evolution of septic shock.
(DOCX)

Table S4 Evolution of SOFA scores.
(DOC)
