# Supplementary figures and images for: Changes in Circulating Procalcitonin Versus C-Reactive Protein in Predicting Evolution of Infectious Disease in Febrile, Critically Ill Patients
Source: PLoS One. 2013 Jun 6;8(6):e65564. doi: 10.1371/journal.pone.0065564 (PMC3675153; doi:10.1371/journal.pone.0065564)

Figure S1. Consort diagram

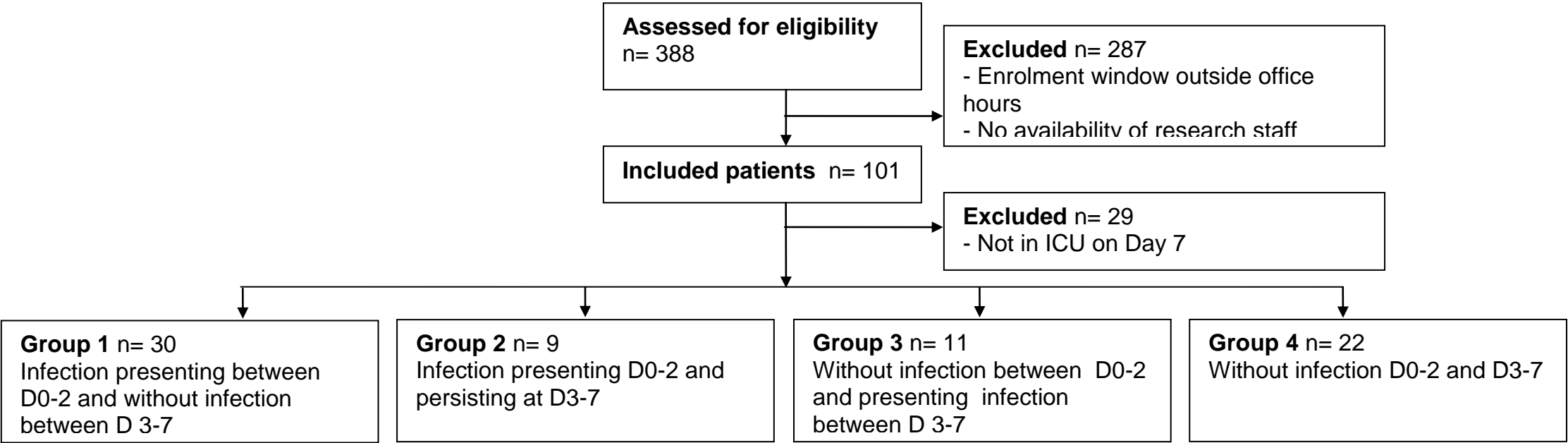

Supplement: Figure S1 — Consort diagram. (PDF) [file pone.0065564.s001.pdf]
