# Supplementary material for: Changes in Circulating Procalcitonin Versus C-Reactive Protein in Predicting Evolution of Infectious Disease in Febrile, Critically Ill Patients
Source: PLoS One. 2013 Jun 6;8(6):e65564. doi: 10.1371/journal.pone.0065564 (PMC3675153; doi:10.1371/journal.pone.0065564)
Supplement: Table S1 — Infection characteristics. (DOCX) [file pone.0065564.s002.docx]

| **Table S1. Infection characteristics.** | | | |  | |  | |
| --- | --- | --- | --- | --- | --- | --- | --- |
|  | | Group 1 | Group 2 | Group 3 | | P | |
|  | | n=30 | n=9 | n=11 | |  | |
| **Infection D0-2** | |  |  |  | |  | |
| Tracheobronchitis | | 8 (27) | 5 (55) | - | | 0.11 | |
| CAP | | 1 (3) | 0 (0) | - | | 0.58 | |
| VAP | | 6 (20) | 1 (11) | - | | 0.54 | |
| Aspiration pneumonia | | 1 (3) | 0 (0) | - | | 0.58 | |
| Pleurisy/empyema | | 1 (3) | 1 (11) | - | | 0.35 | |
| Sinusitis | | 5 (17) | 1 (11) | - | | 0.19 | |
| Catheter infection | | 2 (7) | 2 (22) | - | | 0.18 | |
| Peritonitis | | 2 (7) | 1 (11) | - | | 0.43 | |
| Pancreatitis | | 2 (7) | 0 (0) | - | | 0.43 | |
| Skin and soft tissue | | 7 (23) | 0 (0) | - | | 0.15 | |
| **D3-7** | |  |  |  | |  | |
| Tracheobronchitis | | - | 3 (33) | 2 (18) | | 0.44 | |
| VAP | | - | 1 (11) | 4 (36) | | 0.19 | |
| Aspiration pneumonia | | - | 0 (0) | 0 (0) | | 1.0 | |
| Pleurisy/empyema | | - | 1 (11) | 0 (0) | | 0.26 | |
| Sinusitis | | - | 1 (11) | 2 (18) | | 0.66 | |
| Catheter infection | | - | 1 (11) | 1 (9) | | 0.88 | |
| Peritonitis | | - | 1 (11) | 0 (0) | | 0.26 | |
| Skin and soft tissue | | - | 0 (0) | 4 (36) | | 0.04 | |
| Meningitis | | - | 1 (11) | 0 (0) | | 0.26 | |
| **Local microbiology D0-2** | | |  |  | |  | |
| Enterobacteriaceae | | 9 (30) | 4 (44) | - | | 0.85 | |
| Staphylococci | | 10 (33) | 3 (33) | - | | 1.00 | |
| Pseudomonadaceae | | 5 (17) | 0 (0) | - | | 0.19 | |
| Enterococci | | 4 (13) | 0 (0) | - | | 0.25 | |
| Xantomonadaceae | | 3 (10) | 2 (22) | - | | 0.34 | |
| Yeasts | | 5 (17) | 3 (33) | - | | 0.28 | |
| Miscellaneous | | 12 (40) | 3 (33) | - | | 0.72 | |
| **D3-7** | |  |  |  | |  | |
| Enterobacteriaceae | | - | 4 (44) | 4 (36) | | 0.71 | |
| Staphylococci | | - | 3 (33) | 3 (27) | | 0.80 | |
| Pseudomonadaceae | | - | 0 (0) | 1 (9) | | 0.35 | |
| Enterococci | | - | 0 (0) | 2 (18) | | 0.18 | |
| Xantomonadaceae | | - | 2 (2) | 2 (18) | | 0.82 | |
| Yeasts | | - | 3 (33) | - | | 0.04 | |
| Miscellaneous | | - | 3 (33) | 2 (18) | | 0.82 | |
| **Blood stream infection D0-2** | | |  |  | |  | |
| Enterobacteriaceae | | 1 (3) | 1 (11) | - | | 0.35 | |
| Staphylococci | | 4 (13) | 0 (0) | - | | 0.25 | |
| Enterococci | | 1 (3) | 1 (11) | - | | 0.35 | |
| Yeasts | | 0 (0) | 2 (22) | - | | 0.008 | |
| Miscellaneous | | 1 (3) | 0 (0) | - | | 0.58 | |
| **D3-7** | |  |  |  | |  | |
| Enterobacteriaceae | | - | 1 (11) | 1 (9) | | 0.88 | |
| Staphylococci | | - | 0 (0) | 2 (18) | | 0.18 | |
| Enterococci | | - | 1( 11) | 2 (18) | | 0.66 | |
| Yeasts | | - | 2 (22) | 0 (0) | | 0.10 | |
| Number (percentage); CAP=community-acquired pneumonia; VAP=ventilator-acquired pneumonia; Group 1= infection (I) Day (D)0-2 not D3-7; Group 2= I D0-2 and I D3-7; Group 3= no I D0-2 but D3-7. | | | | | | | |
