# Supplementary material for: Changes in Circulating Procalcitonin Versus C-Reactive Protein in Predicting Evolution of Infectious Disease in Febrile, Critically Ill Patients
Source: PLoS One. 2013 Jun 6;8(6):e65564. doi: 10.1371/journal.pone.0065564 (PMC3675153; doi:10.1371/journal.pone.0065564)
Supplement: Table S2 — Evolution of bloodstream infection. (DOCX) [file pone.0065564.s003.docx]

| **Table S2. Evolution of bloodstream infection.** | | | | | |
| --- | --- | --- | --- | --- | --- |
|  | Group 1a | Group 2a | Group 3a | Group 4a | p |
|  | n=8 | n=2 | n=5 | n=57 |  |
| WBC D0-2, x10^9^/L | 18.2 (2.5-27.5) | 53.2 (24.7-81.7) | 13.9 (9.0-19.8) | 12.8 (7.8-24.8) | 0.08 |
| WBC D7, x10^9^/L | 15.5 (8.0-23.2) | 24.2 (18.3-30.2) | 23.1 (10.5-33.0) | 12.3(4.9-29.2) | 0.007 |
| WBC change | 0.76 (0.55-3.20) | 0.56 (0.37-0.74) | 1.17 (1.14-2.95) | 0.88(0.40-2.33) | 0.02 |
| CRP D0-2, mg/L | 220 (71-397) | 362 (303-421) | 139 (38-257) | 183 (5-440) | 0.07 |
| CRP D7, mg/L | 57 (3-267) | 205 (22-389) | 54 (101-304) | 85 (2-416) | 0.20 |
| CRP change | 0.14 (0.04-1.01) | 0.50 (0.07- 0.92) | 1.07 (0.73-6.97) | 0.55 (0.02-2.93) | 0.02 |
| PCT D0-2, ng/mL | 1.6 (0.09-45.1) | 74.2 (73.2-75.3) | 0.8 (0.3-3.4) | 0.6 (0.08-37.2) | 0.07 |
| PCT D7, ng/mL | 0.2 (0.06-7.8) | 13.6 (2.9-24.3) | 2.1 (1.3-20.8) | 0.2 (0.06-38.5) | 0.002 |
| PCT change | 0.32 (0.05-2.97) | 0.19 (0.04-0.33) | 2.80 (0.45-68.3) | 0.43 (0.05-5.88) | 0.01 |
| Lactate D0-2, mmol/L | 1.7 (1.1-3.5) | 2.6 (1.8-3.5) | 1.2 (1.0-1.5) | 1.4 (0.5-2.3) | 0.05 |
| Lactate D7, mmol/L | 2.1 (1.2-4.3) | 2.5 (1.8-3.1) | 1.3 (1.1-1.6) | 1.0 (0 -2.2) | 0.002 |
| Lactate change | 1.15 (0.50-2.08) | 1.12 (0.51-1.72) | 1.01(0.73-1.60) | 0.79 (0-1.50) | 0.20 |
| Median (range) for WBC=white blood cell count; CRP=C-reactive protein; PCT=procalcitonin. Group 1a=bloodstream infection (BSI) Day (D) 0-2 not D3-7; Group 2a= BSI D0-2 and BSI D3-7; Group 3a= no BSI D0-2 but D3-7; Group 4a= no BSI D0-2 nor D3-7. | | | | | |
