# Supplementary material for: Changes in Circulating Procalcitonin Versus C-Reactive Protein in Predicting Evolution of Infectious Disease in Febrile, Critically Ill Patients
Source: PLoS One. 2013 Jun 6;8(6):e65564. doi: 10.1371/journal.pone.0065564 (PMC3675153; doi:10.1371/journal.pone.0065564)
Supplement: Table S3 — Evolution of septic shock. (DOCX) [file pone.0065564.s004.docx]

| **Table S3. Evolution of septic shock.** | |  |  |  |  |
| --- | --- | --- | --- | --- | --- |
|  | Group 1b | Group 2b | Group 3b | Group 4b | p |
|  | n = 16 | n = 5 | n = 10 | n = 41 |  |
| WBC D0-2, x10^9^/L | 13.9 (2.5-24.4) | 19.8 (7.9-81.7) | 17.5 (9.0-27.5) | 12.3 (7.8-23.9) | 0.17 |
| WBC D7, x10^9^/L | 14.5 (4.9-23.2) | 17.1 (9.3-30.2) | 19.0 (10.5-33.0) | 10.7 (5.3-29.2) | 0.001 |
| WBC change | 0.93 (0.47-3.20) | 0.74 (0.37-1.34) | 1.14 (0.60-2.95) | 0.81(0.40-2.33) | 0.25 |
| CRP D0-2, mg/L | 243 (5.0-397) | 306 (102-421) | 142 (38-257) | 181 (5-440) | 0.004 |
| CRP D7, mg/L | 57 (3.0-416) | 182 (22-389) | 156 (101-304) | 61 (2-265) | 0.01 |
| CRP change | 0.31 (0.04-1.12) | 0.56 (0.07-1.82) | 1.03 (0.48-6.97) | 0.51 (0.02-2.58) | 0.003 |
| PCT D0-2, ng/mL | 1.1(0.08-45.1) | 8.2 (0.3-75.3) | 1.3 (0.08-6.3) | 0.5 (0.09-37.1) | 0.02 |
| PCT D7, ng/mL | 0.2 (0.06-2.6) | 0.6 (0.2-24.3) | 1.6 (0.2-20.8) | 0.2 (0.06-3.5) | 0.001 |
| PCT change | 0.18 (0.05-2.00) | 0.18 (0.04-0.64) | 1.73 (0.19-68.3) | 0.43 (0.08-5.88) | <0.001 |
| Lactate D0-2, mmol/L | 1.6 (1.0-3.5) | 1.5 (1.0-3.5) | 1.2 (0.9-2.3) | 1.4 (0.5-2.2) | 0.32 |
| Lactate D7, mmol/L | 1.2 (0-4.3) | 1.3 (0.9-3.1) | 1.1 (0.7-2.4) | 1.0 (0.5-2.7) | 0.21 |
| Lactate change | 0.86 (0-1.23) | 0.83 (0.51-1.72) | 1.00 (0.43-1.60) | 0.80 (0.38-2.08) | 0.69 |
| Median (range) for WBC=white blood cell count; CRP=C-reactive protein; PCT=procalcitonin. Group 1b=septic shock (SS) Day (D) 0-2 not D3-7; Group 2b= SS D0-2 and SS D3-7; Group 3b= no SS D0-2 but D3-7; Group 4b=no SS D0-2 nor D3-7. | | | | | |
