# Supplementary material for: Changes in Circulating Procalcitonin Versus C-Reactive Protein in Predicting Evolution of Infectious Disease in Febrile, Critically Ill Patients
Source: PLoS One. 2013 Jun 6;8(6):e65564. doi: 10.1371/journal.pone.0065564 (PMC3675153; doi:10.1371/journal.pone.0065564)
Supplement: Table S4 — Evolution of SOFA scores. (DOC) [file pone.0065564.s005.doc]

| **Table S4. Evolution of SOFA scores.** | |  |  |  |
| --- | --- | --- | --- | --- |
|  | Group 1c | Group 2c | Group 3c | p |
|  | n = 52 | n = 8 | n = 8 |  |
| WBC D0-2, x10^9^/L | 13.5 (2.5-27.5) | 13.2 (9.2-19.8) | 16.1(8.0-81.7) | 0.76 |
| WBC D7. x10^9^/L | 13.0 (4.9-33.0) | 13.2 (8.5-27.2) | 15.5 (6.9-30.2) | 0.37 |
| WBC change | 0.83 (0.40-3.20) | 1.09 (0.67-1.96) | 0.82 (0.37-2.56) | 0.30 |
| CRP D0-2. mg/L | 202 (5-440) | 173 (38-290) | 157 (59-421) | 0.87 |
| CRP D7. mg/L | 85 (2-416) | 160 (41-304) | 92 (18-389) | 0.13 |
| CRP change | 0.51 (0.02-2.93) | 0.95 (0.20-6.97) | 0.72 (0.17-1.03) | 0.11 |
| PCT D0-2, ng/mL | 0.67 (0.08-75.3) | 0.68 (0.08-1.98) | 1.42 (0.14-73.2) | 0.37 |
| PCT D7. ng/mL | 0.23 (0.06-38.5) | 0.49 (0.15-10.4 | 1.36 (0.12-24.3) | 0.13 |
| PCT change | 0.39 (0.04-68.3) | 1.73 (0.18-5.88) | 0.67 (0.18-1.79) | 0.01 |
| Lactate D0-2, mmol/L | 1.4 (0.5-3.5) | 1.2 (0.50-1.8) | 1.7 (1.2-3.5) | 0.03 |
| Lactate D7, mmol/L | 1.1 (0.5-2.7) | 1.1 (0.7-1.9) | 1.0 (0.6-4.3) | 0.96 |
| Lactate change | 0.80 (0.38-2.08) | 1.17 (0.77-1.40) | 0.63 (0.38-1.72) | 0.15 |
| Median (range) for WBC=white blood cell count; CRP=C-reactive protein; PCT=procalcitonin. Group 1c decreasing SOFA scores between D0-2 and D7. Group 2c unchanged SOFA scores, Group 3c increase in SOFA scores. | | | | |
